# Abatacept induced granulomatous hepatitis with a sarcoidosis- like reaction: a blinded trial in mice

**DOI:** 10.1186/s40360-019-0303-0

**Published:** 2019-05-07

**Authors:** Sultan M. Almogairen

**Affiliations:** Rheumatology Division, Department of Medicine, College of Medicine, King Saud University, P O Box 2925, Riyadh, 11461 Saudi Arabia

**Keywords:** Abatacept, Granuloma, Hepatitis, Sarcoidosis

## Abstract

**Background:**

Abatacept is increasingly used for rheumatoid arthritis (RA) and juvenile idiophathic arthritis (JIA) treatment. However little is known about the risk of hepatotoxicity. The aim of this study was to determine whether the inhibition of the T cell CD28 receptor by abatacept results in acute hepatitis in BALB/c mice.

**Methods:**

Twenty BALB/c mice were studied. Ten mice received subcutaneous (SC) injection of abatacept (0.25mg per 25g body weight per 0.03 ml normal saline) at 0, 2, 4 and 8 weeks. For the control group, 10 mice received a SC injection of normal saline (NS) (0.03 ml). At the 10th week post injection, the mice were sacrificed, and histopathological studies were conducted.

**Results:**

Of the abatacept-treated group, 3/10 mice died. Liver histology for the abatacept-treated group showed that 6/7 displayed histopathological changes in the lobular cellular infiltrates of eosinophils, lymphocytes and histiocytes, in addition to granuloma formation. In contrast, only minimal inflammation was observed in 3/10 mice in the control group (*p*=0.036).

**Conclusion:**

Abatacept may play a role in inducing granulomatous hepatitis with a sarcoidosis-like reaction. Additional data including transaminases, antinuclear antibodies (ANA), Antimitochondrial antibodies (AMA) and other auto antibodies should be tested.

## Background

Rheumatoid arthritis (RA) treatments have evolved in the last 20 years with the widespread usage of methotrexate, followed by the development of Tumor necrosis factor (TNF) alpha inhibitors [1, 2]. Patients who fail Tumor Necrosis Factor Inhibitor (TNFi) therapy can switch to another TNFi (TNFi cyclers) or to a non-TNFi drug, such as abatacept, rituximab, tocilizumab. Alternatively, patients can also be prescribed the targeted synthetic Disease-Modifying Anti Rheumatic Drugs (DMARD) tofacitinib (non-TNFi switchers) [3–8].

In the early 1990s, Linsley et al. synthesized a fusion protein using a human IgG1 and a modified Fc region of Cytotoxic T Lymphocyte Antigen-4 (CTLA4), which was capable of inhibiting the immune response in vitro. This protein was originally known as the CTLA4-Ig and it was subsequently named abatacept [9, 10]. Abatacept binds to the CD80/86 receptor of an antigen-presenting cell. This interaction blocks the activation of the CD28 receptor on T cells [11, 12].

In the global safety database overall frequencies of adverse events (AEs; 88.8% versus 85.1%), serious AEs (SAEs; 14.0% versus 12.5%) and malignancies (1.4% versus 1.1%) were statistically similar in abatacept-treated-versus placebo-treated patients, respectively. This was found to be the case regardless of the potential relationship between the study therapies. Discontinuation of the drug therapy as a result of SAEs was 2.8% in the abatacept group versus 1.6% in the placebo group. The frequency of serious infections was low overall (3.0% versus 1.9%) in the abatacept-treated versus placebo-treated patients, respectively [13, 14].

Abatacept has no known hepatotoxic effects except for causing an elevation in alanine transaminase and aspartate transaminase levels, which were reported in forty six out of eighteen hundred and seventy nine (46/1879) patients (2.4%) and 26/1879 patients (1.4%), respectively. There are no hepatic contraindications for abatacept treatment except for severe liver failure. This limits the use of all drugs, and pharmacokinetic and pharmacodynamic studies are required to define the adverse effects of abatacept use, if any [15, 16].

Compared to the number of human studies, there are no controlled experimental animal studies that have examined the histology of abatacept-induced liver injury. Therefore, this study determined the effect of blocking the cluster of differentiation 28/ cytotoxic T-lymphocyte-associated antigen 4/B7 (CD28/CTLA-4/B7) costimulatory pathway by SC injection of abatacept with respect to the development of acute hepatitis in BALB/c mice.

## Methods

Twenty BALB/c male mice (average weight of 25g and they were not previously subjected to therapy or experiments) were purchased from the Faculty of Pharmacy at King Saud University (Kingdom of Saudi Arabia). This study was approved by King Saud University, Faculty of Medicine Institutional review board (IRB) through the institution's research ethics board (REB) approval number (E-14-1300). This research was conducted in compliance with the research ethics standards. All mice received humane care and that study protocols comply with the institution's guidelines.

Mice were kept in polycarbonate metrolon plastic cages covered with a stainless-steel cover in the animal house at the College of Medicine at King Saud University in Riyadh, Saudi Arabia. The mice were exposed to 12h of darkness and 12h of light daily, and were kept under observation for 3 weeks. No evidence of sickness was observed. All mice were 12-14 weeks old at the start of the experiment.

This is a pilot study involving twenty mice simply divided into two groups that each consisted of ten mice. The unit of analysis for each dataset was group of animals. One group received SC injections of abatacept (0.25mg/ 0.03 ml NS) at 0, 2, 4 and 8 weeks. The second group received a SC injection of normal saline (0.03 ml) weekly. We used weekly NS injection for this common control group because this study is considered as a part of multi-arm animal trials.

Since the appropriate dosage for SC injections of abatacept has not been reported in animals, we extrapolated this based upon recommended doses used in human studies. Ruperto et al.[17] injected children (10-25kg body weight) diagnosed with juvenile idiophathic arthritis with 50mg of abatacept. Thus abatacept sc 50mg for child weighing 10 kg, corresponded to 0.125 mg weekly or 0.25 mg every other week for the average mouse weighing 25g.

During the 10th week, mice were sacrificed by applying pressure to the neck and dislocating the cervical spine. Subsequently liver histopathological studies were performed for each mouse in both groups. The liver tissues were bisected and fixed in 10% buffered formalin for 24h. The tissues were then processed using hematoxylin and eosin (H/E) stain. The slides were examined using a light microscope. Evaluation of liver tissue slides were performed blindly by a histopathologist.

### Statistical Analysis

Statistical differences between the abatacept-treated and saline-treated groups were calculated using Fisher’s exact test. A *p*-value < 0.05 was considered significant.

## Results

Of the abatacept-treated mice, 3/10 died during the last 3 weeks of the experiment. There was no clinical evidence of sickness prior to death. No deaths were reported in the control group.

**Fig. 1:**
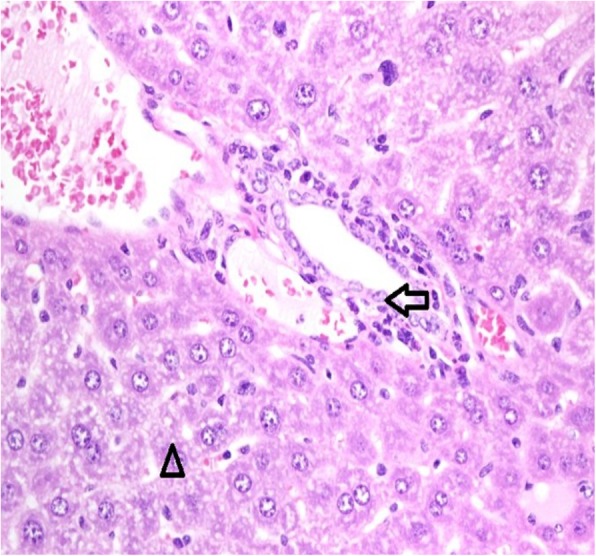
Histology of a normal portal duct (arrow) and liver lobule (arrowhead) in control liver. H/E staining, 400× magnification

**Fig. 2:**
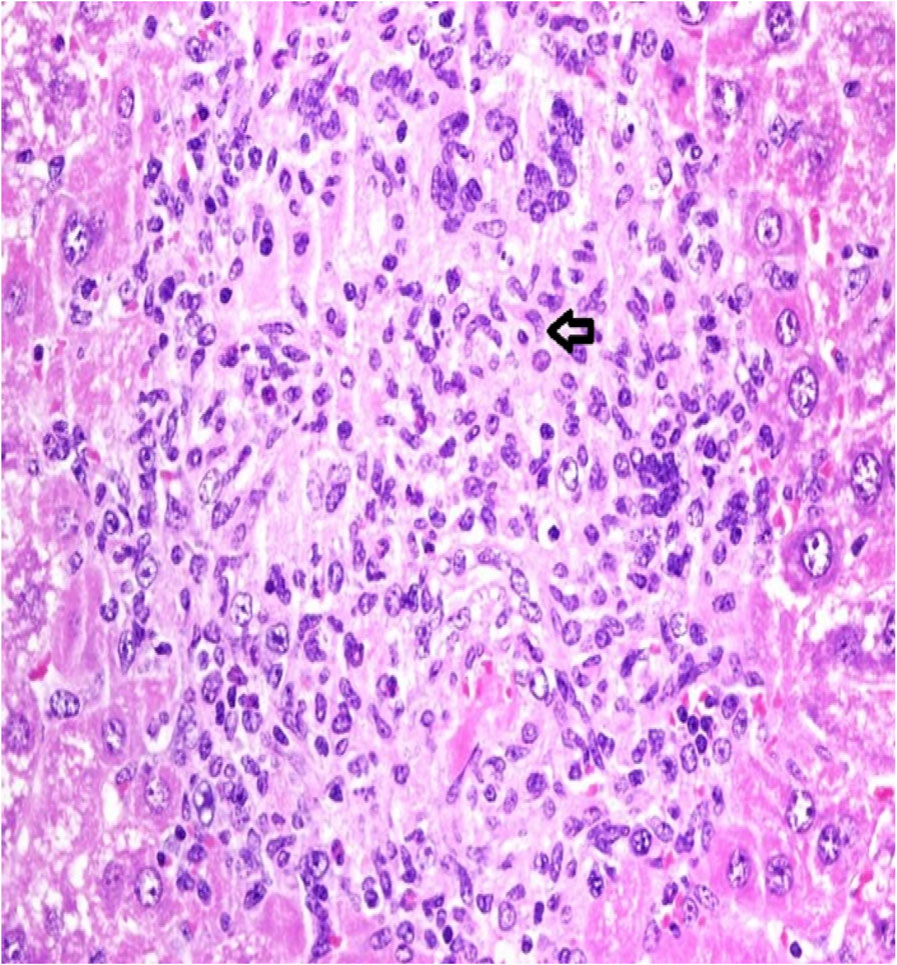
Histology of an abatacept-treated liver reveals well-defined collection of epithelioid histiocytes (arrow) without necrosis. H/E staining, 400× magnification

**Fig. 3:**
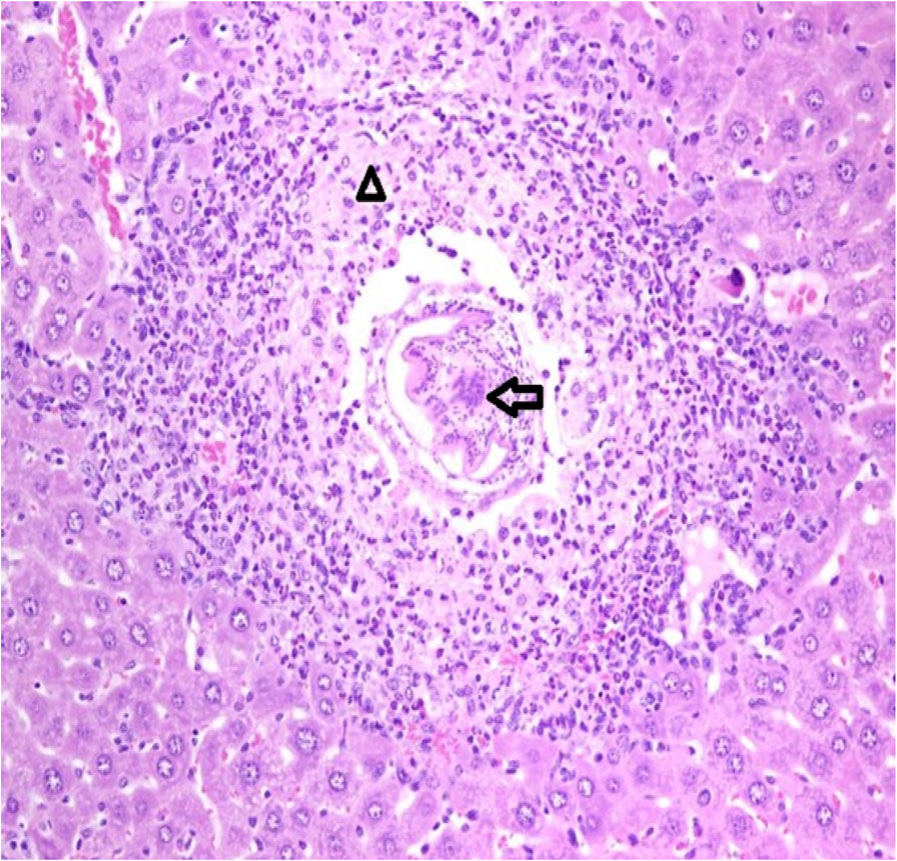
Histology of mouse #S7 control liver reveals epithelioid histiocytes (arrow head) surrounding a parasitic organism (arrow). H/E staining, 200× magnification

Histopathological results are shown for the normal saline control group (Table 1) and the abatacept-treated group (Table 2). Liver changes were negative if all the histological features were normal (Fig. 1). Otherwise they remained positive. Positive liver tissues were observed in 6/7 mice treated with SC injections of abatacept (Table 2 and Fig. 2). In contrast, positive, mild liver changes were observed in 3/10 mice in the control group (*p*=0.036). In two of these animals, the liver displayed lobules with mild inflammation and infiltration by lymphocytes. The liver of the third mouse (#S7 mouse) presented with a parasite and granuloma (Fig. 3).

**Table 1:** Histopathological features of the control group

Mouse No.	Portal Tract	Liver Lobule
S1	Normal	Normal
S2	Normal	Normal
S3	Normal	Lymphocytes +
S4	Normal	Lymphocytes +
S5	Normal	Normal
S6	Normal	Normal
S7	lymphocytes++, histiocytes+	Granuloma and parasite
S8	Normal	Normal
S9	Normal	Normal
S10	Normal	Normal

Mild (+), Moderate (++), Marked (+++)

**Table 2:** Histopathological features of the abatacept-treated group

Mouse No.	Portal Tract	Liver Lobule
B1	Granuloma, Eosinophils ++ lymphocytes++, histiocytes+	Small Granuloma and eosinophils+ Apoptosis
B2	Eosinophils+++, lymphocytes ++, histiocytes+	Small Granuloma and eosinophils++ Apoptosis + Focal EMH+
B3	neutrophils+++, lymphocytes++, histiocytes+	Small Granuloma and eosinophils++ Apoptosis +
B4	Eosinophils+, lymphoctes +, histiocytes+	Granuloma and eosinophils++
B5	Eosinophils+, lymphoctes +, histiocytes+	Granuloma+++ and eosinophils++
B6	Normal	Normal
B7	lymphocytes+ plasma cells, histiocytes+	Focal EMH

EMH (extramedullary hematopoiesis)

Mild (+), Moderate (++), Marked (+++)

## Discussion

Abatacept has recently been approved as a second-line treatment for RA and JIA. However, in systematic lupus erythematosus randomized clinical trials, this drug has failed to achieve its primary outcome [1–9, 11, 13, 18].

Abatacept demonstrated an acceptable safety profile which was also observed in different studies [13, 14]. In contrast, the present study, mortality was observed in 30% of the abatacept-treated mice despite using the recommended dose for JIA (as per body weight category above). Meanwhile, in the control group no mortality was reported. The husbandry was the same for both groups, and there were no clinical signs of sickness prior to death. The deaths were probably the result of cardiovascular events.

Rieke Alten et al. [15] investigated the safety of long-term SC abatacept injections using integrated analysis of clinical trial data for more than 4 years of treatment. Death occurred at an incidence rate of 0.59 (95% confidence interval (CI): 0.40-0.88). Of the seven deaths classified as “certainly”, “probably”, or “possibly” related to the treatment, four were due to infections, one to a peritoneal neoplasm, one to a combination of a wound infection and malignant lung neoplasm, and one to multi-organ failure. An additional 18 deaths were considered unlikely to be related to the treatment. These deaths were most frequently the result of cardiovascular events.

Studies have documented the safety of abatacept in treating RA, including in patients with evidence of viral hepatitis [19–21].On the other hand cases of acute hepatitis B viral infection reactivation have been reported in patients receiving this drug. Therefore, patients should be screened for hepatitis B and C before the start of therapy [22, 23].

For the sake of honesty we reported all the abnormal histopathological finding even in the control. We found only single hepatic granuloma surrounding a parasite of mouse S7 _,_ the background liver parenchyma was unremarkable without significant inflammation. This granuloma include a parasite helminth rather than unicellular protozoa, so it is not a leishmania, and in the absence of eggs it is unlikely to be schistosoma granuloma but probably it is a visceral larva migrans (Toxocariasis (T) ). BALB/c mice seem exceptionally susceptible to *T. canis* as larva counts are much higher compared to other strain. They usually did not show any clinical symptom [24].

Little information is available regarding the changes that occur in the liver following abatacept-treatment [16, 18–21]. Iwanaga N et al. [16] reported the occurrence of severe liver injury in abatacept-treated RA patient without reactivation of hepatitis B virus.

In the present study abatacept treated mice displayed significant histopathological changes in the liver (*p*=0.036) with respect to lobular cellular infiltration of eosinophils, lymphocytes, histiocytes with apoptosis and small granuloma formation.

Hepatic injury occurs as a result of different processes, including direct injury or autoimmunity. Since lobular inflammation and infiltration of eosinophils, histiocytes and lymphocytes with granuloma were observed in the absence of the characteristic histological features of autoimmune hepatitis including interface hepatitis, lymphocytic/ lymphoplasmacytic infiltrate without eosinophils presence [25–27], rarely granuloma are seen [28]. So most likely diagnosis is Abatacept induced granulomatous hepatitis but probably an overlapping syndrome could not be excluded [29–32].

We cannot rule out the possibility of autoimmune hepatitis unless the abatacept treated mice do not meet the simplified diagnostic criteria (2008). According to the simplified diagnostic criteria (2008) of the international autoimmune hepatitis group, selective elevation of IgG with autoantibodies is a hallmark of autoimmune hepatitis. These autoantibodies include ANA, anti-soluble liver antigen/liver-pancreas smooth-muscle antibodies (SMA), antibodies to liver-kidney microsomes (LKM) anti-soluble liver antigen/ liver-pancreas (SLA-LP) autoantibodies [33].

Granulomas are aggregates of modified macrophages (epithelioid cells) and other inflammatory cells that accumulate after chronic exposure to antigens so presence of granuloma in the absence of fibrosis probably more in favor subacute rather than chronic hepatitis [34].

Sarcoidosis-like reactions have been reported after treatment with TNF alpha blockade drugs [31, 32, 35], However, so far, no evidence in the literature to indicate that abatacept causes granulomatous hepatitis in humans, but probably because majority of patients with drug induced hepatic granuloma are asymptomatic and 60% of them are reported to have elevated transaminases but did not meet the criteria for liver biopsy. These will indicate the contrast between the limited liver injury in humans discovered by high transaminases and the findings of the current study [36–38]. Previous literature does not reflect the magnitude of drug–induced granulomatous hepatic disease and that many cases reported as “granulomatous hepatitis” consistent with “sarcoidosis” as well as many “undiagnosed” cases have a drug etiology. There have recently been reports of hepatic granulomas induced by drugs that had not previously been considered to be causal of this condition, and we therefore believe that many more drugs may potentially play a role in the development of hepatic granuloma [34, 39, 40].

Necrotizing granulomas in infectious disease processes often do not respect the architecture of the liver and may destroy adjacent structures. Necrotizing epithelioid granulomas quite frequently have an infectious etiology, and associated with Supportive inflammation .On the other hand necrotizing granuloma rarely induced by drugs. So it is unlikely that hepatic granuloma in Abatacept treated group is due to infection in immunocompromised mice [41].

## Conclusion

To our knowledge this is the first control blinded study of BALB/c mice that has demonstrated granulomatous allergic hepatitis with sarcoidosis-like reaction following SC injections of abatacept.

Further experimental and clinical studies with transaminases, ANA, antimitochondrial antibodies (AMA) and serum-specific markers of autoimmune hepatitis are needed to determine the mechanisms underpinning abatacept-induced hepatitis.

Special histological stains, including the Ziehl-Neelsen (Zn) stain and fungal Grocott-Gomori’s / Periodic acid-Schiff (GMS/-PAS) stains, are needed to better assess the granulomatous inflammatory reaction and rule out tuberculosis and fungal infections

## Abbreviations

AE: Adverse events
ANA: Anti-nuclear antibodies
CD: Cluster of Differentiation
CI: Confidence interval
CTLA4: Cytotoxic T Lymphocyte Antigen-4
DMARD: Disease-Modifying Anti Rheumatic Drugs
GMS/-PAS: Grocott-Gomori’s / Periodic acid-Schiff
H/E: Hematoxylin and eosin
IgG: Immunoglobulin
JIA: Juvenile idiophathic arthritis
LKM: Liver-kidney microsomes
NS: Normal saline
RA: Rheumatoid arthritis
SAE: Serious Adverse events
SC: Subcutaneous
SLA-LP: Soluble liver antigen/ liver-pancreas autoantibodies
SMA: Smooth-muscle antibodies
TNFi: Tumor Necrosis Factor alpha Inhibitor
Zn: Ziehl-Neelsen
